# Rather a versatile multi-tool than a sword: an integral role of the plasminogen system in health and disease

**DOI:** 10.3389/fimmu.2026.1878106

**Published:** 2026-07-03

**Authors:** Rostislav Skrabana, Maja Castven, Maria Tomková, Erik Sedlák, Patrik Babulic, Michaela Jakubcova, Tetiana Moskalets, Vladimir Leksa

**Affiliations:** 1 Laboratory of Structural Biology of Neurodegeneration, Institute of Neuroimmunology, Slovak Academy of Sciences, Bratislava, Slovakia; 2 Laboratory of Molecular Immunology, Institute of Molecular Biology, Slovak Academy of Sciences, Bratislava, Slovakia; 3 Center for Interdisciplinary Biosciences, P. J. Šafárik University in Košice, Košice, Slovakia; 4 Department of Biochemistry, Faculty of Science, P. J. Šafárik University in Košice, Košice, Slovakia

**Keywords:** amyloidosis, cancer, fibrinolysis, homeostasis, infection, inflammation, plasminogen, proteolysis

## Abstract

Proteolysis, the irreversible, hydrolytic cleavage of peptide bonds by proteases, is essential for life. The plasminogen system, one of the central proteolytic systems, regulates diverse physiological pathways, including fibrinolysis, inflammation, wound healing, and tissue remodelling. Beyond its proteolytic functions, the plasminogen system serves as a hub for crosstalk to maintain homeostasis. Yet, its dysregulation, misuse, or hijacking by pathogens can drive pathologies such as hereditary disorders, tumour dissemination, bacterial invasion, and viral priming. This review explores its evolution, structural aspects, activation mechanisms, regulatory pathways, and pharmacological modulation of the plasminogen system, synthesising decades of research with recent advances. We highlight the multifaceted nature of the plasminogen system—as both a guardian of physiological balance and a potential driver of disease—and discuss its components as therapeutic targets and tools.

## Introduction

The genes encoding proteases represent more than 2% of the human genome (1); it is therefore no wonder that proteolysis plays a crucial role in all biological processes (2). On the other hand, 100% of genes encode potential protease substrates, and even small changes in these genes can have large consequences for proteolytic pathways and, by extension, health.

In 2025, Tushir-Singh and colleagues revealed that, compared with chimpanzees and other primates, the cell-death regulator Fas Ligand (FasL) in humans contains a Pro153-Ser153 evolutionary substitution, rendering human FasL more susceptible to cleavage by the serine protease plasmin. Since FasL on activated cytotoxic T cells is critical for killing tumour cells, its removal by plasmin contributes to the higher risk of humans developing cancer than our closest living ancestors, the nonhuman primates. Despite sharing >98% genomic similarity (3).

The plasminogen activation system, in which plasmin plays a central role, has long been recognised as a pathway specifically responsible for fibrin clot dissolution, counterbalancing the coagulation system, and also for endowing cells with migratory potential. However, during the last decade, it has become more and more evident that the plasminogen system plays a multitude of cross-talk functions, some of which are even independent of proteolytic activity, which interconnect various molecular pathways, for example, in signalling, activation of growth factors, macrophage reprogramming, neutrophil apoptosis, efferocytosis, wound healing, or inflammation resolution (4–13). On the other hand, as clearly described in the human FasL case above, the plasminogen activation system contributes not only to homeostasis but also to many disorders, such as cancer, infections, and neurodegenerative disorders, making components of the plasminogen system attractive targets for therapy (3, 11, 14, 15).

**Figure 1:**
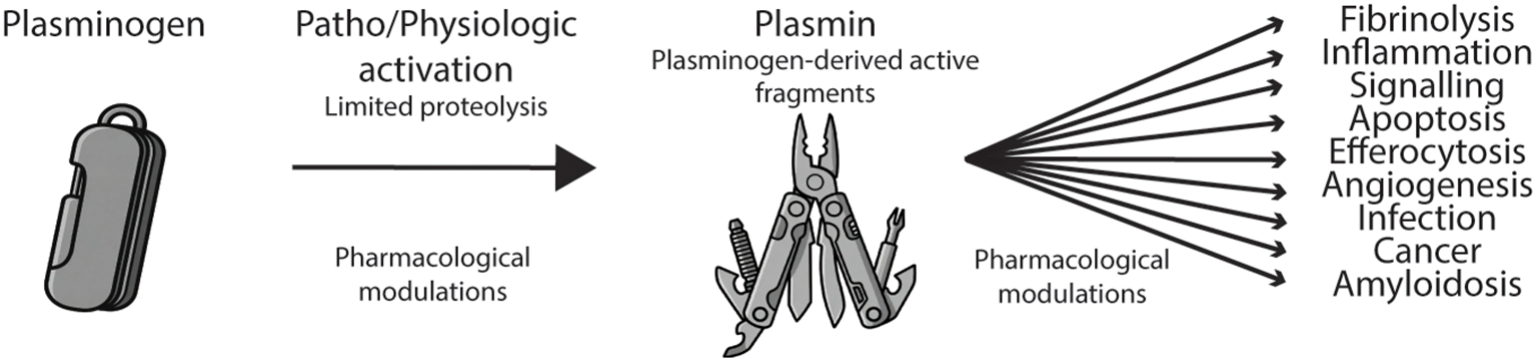
Schematic representation of the Plg system. The Plg system is best known for fibrin clot dissolution and cell migration. Recent evidence reveals its broader roles, including growth factor signalling, apoptosis, efferocytosis, angiogenesis, or inflammation resolution. It also contributes to various diseases, including cancer, infections, and amyloidosis. Altogether, it makes components of the Plg system attractive therapeutic targets and tools. This review provides an updated overview of the Plg system, focusing on its evolution, structure, functions, activation mechanisms, regulation, pathophysiological relevance, and pharmacological modulation.

Therefore, the plasminogen system should no longer be regarded merely as a scissor or a double-edged sword, but rather as a versatile multi-tool. This review offers a comprehensive and updated perspective on the plasminogen system, highlighting recent advances in our understanding of its evolution, structure, functions, activation mechanisms, regulatory pathways, pathophysiological relevance, and pharmacological modulation (**Figure 1**).

## Classification, evolution and structure of plasminogen

Plasminogen (Plg), a central enzyme of the plasminogen system, was first described in the mid-20th century as a blood component that might be converted by streptococcal cultures to the active form, originally termed “fibrinolysin”, that dissolved fibrin clots (16). It was purified from blood plasma, which had given it its name ultimately (17).

**Figure 2:**
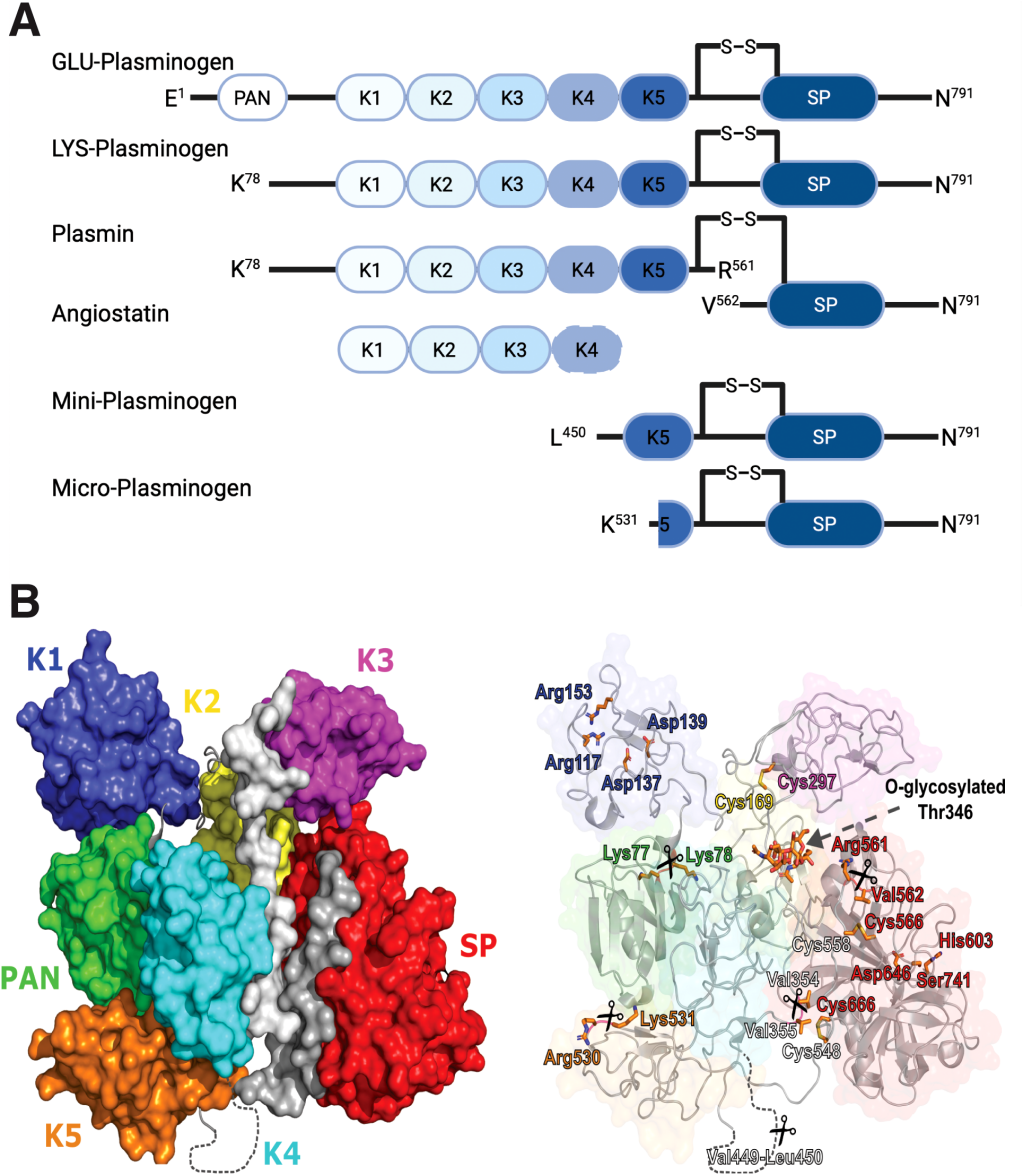
Plg structure. **(A)** Schematic structure of Plg and its derived molecules. The figure illustrates the modular structure of GLU-plasminogen (Glu-Plg), LYS-plasminogen (Lys-Plg), plasmin, miniplasminogen, microplasminogen, and angiostatin, highlighting their functional domains: the plasminogen–apple–nematode domain (PAN), the kringle domains (K1–K5), and the proteolytic domain (SP). Corresponding residues and disulfide bridges are depicted. Figure was created with BioRender.com. **(B)** Insights into Plg conformation from the available X-ray structural studies of the Glu-Plg. Plg glycoforms were crystallised, yielding three closed form configurations with PDB ID 4DUR, 4DUU (23) and 4A5T (27). The main features of closed Plg are shown in the structure 4A5T. A space-filling model (left panel) shows a tight association of individual domains of the closed Plg conformation (PAN, green; SP, red; K1 – K5, blue, yellow, violet, cyan and orange, respectively) interconnected by linkers of various lengths. (cartoon representation in grey). In addition, a cartoon model (right panel) highlights the position of some important structural features with sticks. Four N-terminal domains are tightly arranged by 3–4 amino acid linkers (PAN-K1, K1-K2) or by an interdomain disulphide bond (K2-K3; Cys169-Cys297). PAN-K1 linker is preceded by the scissile bond Lys77-Lys78 for the creation of Lys-Plg. Arg117, Asp137, Asp139 and Arg153 form the LBS in K1, the only one available for lysine binding in the closed form of Plg. The remaining linkers are longer; the K3-K4 linker is 24-amino-acid long (light grey space filling), contains the ubiquitous O-glycosylated Thr346 (stick representation), and a scissile bond between Val354 and Val355, producing K1-K3 angiostatin. The 28-amino acid-long K4-K5 linker (dashed line) is flexible and completely disordered in all three available Plg structures. It contains a scissile bond between Val449 and Leu450, producing K1-K4 angiostatin and mini-Plg. The scissile bond for micro-Plg formation lies between the K5 surface residues Arg530 and Lys531. Finally, K5 and SP are connected by a 21-amino acid-long activation linker (dark grey space-filling) containing the scissile bond Arg561-Val562, which is cleaved during Plg activation by plasminogen activators, and the catalytic triad Ser741-His603-Asp646 adopts the active orientation. Upon activation, the SP domain remains connected to the rest of the molecule via two disulfide bonds, Cys548-Cys666 and Cys558-Cys566, within the activation linker. Domain limits are as annotated in the UniProt entry P00747 (PLMN_HUMAN).

Plg is a zymogen, an inactive precursor that is proteolytically processed by Plg activators, tissue-type plasminogen activator (tPA), and urokinase-type plasminogen activator (uPA), which cleave the activation loop at Arg561-Val562 peptide bond to generate the active serine protease plasmin (**Figures 2A, B**). Many other proteases, including plasmin itself, may process Plg, yielding functional molecules, notably Lys-plasminogen, angiostatins, mini-plasminogen (miniPlg), and microplasminogen (microPlg). Plasmin belongs to the S1 trypsin-like family of serine proteases, also known as the peptidase S1 family. The peptidase S1 family is one of the largest protease groups and includes trypsin, chymotrypsin, granzyme B, hepatocyte growth factor activator (HGFA), elastase, plasma and tissue kallikreins, complement components, and many other proteases. Based on the conserved functional features, there is significant homology among S1 family proteases within the active site, i.e. the substrate-binding pocket of the protease domain and the catalytic triad Ser-His-Asp. Their homology might have arisen from their shared evolutionary origin (18).

Plg-like proteins are found in basal chordates and early vertebrates. In mammals, Plg and its activators diversified. The *PLG* gene likely arose through gene duplication from a common ancestor shared with prothrombin and other related proteases. This duplication enabled the evolution of specialised functions, e.g., fibrinolysis versus coagulation, suggesting that the Plg system evolved alongside the coagulation system to regulate clot formation and dissolution. After duplication, the Plg gene acquired additional segments through exon shuffling or domain duplication, resulting in its unique structure and peculiar functions (19).

Interestingly, the apolipoprotein(a) (Lp(a)) is believed to have arisen from a duplication of the *PLG* gene, followed by loss of the protease domain and expansion of kringle domains (20, 21). Moreover, the *LPA* gene is located on the chromosome region 6q26, very close to the *PLG* gene. Also, the *IGF2R* gene encoding the mannose 6-phosphate/insulin-like growth factor 2 receptor (M6P/IGF2R, CD222), known to internalise the Plg molecule (4), is closely linked to the *PLG* gene in both humans and mice (22). The evolutionary proximity of these genes indicates functional interconnections, as described below.

Human Plg is secreted as a polypeptide with a molecular weight of 89–92 kDa. Plg is ubiquitously O-glycosylated at Thr346 (Plg glycoform II), and about 40% of circulating Plg is also N-glycosylated at Asn289 (Plg glycoform I). The sugar chains at Asn289 interfere with intramolecular Plg contacts, thereby increasing the flexibility of glycoform I (23). At the N-terminus lies the plasminogen–apple–nematode domain, also known as the PAN module (Pan-Apple, PAp). The crystal structure of the full-length Plg revealed that the PAN module, through interaction with the adjacent kringle domains, maintains the closed, activation-resistant conformation of the circulating zymogen (23). The PAN module is followed by five disulfide-formed kringle domains K1–K5. All kringles except K3 contain lysine-binding sites that mediate interactions of Plg with cell-surface receptors or with extracellular matrix proteins (ECM). The C-terminal serine protease domain (SP) encompasses the canonical His–Asp–Ser catalytic triad and characteristic fold of trypsin-like serine proteases (**Figures 2A, B**).

The native full-length Plg, termed Glu-plasminogen (Glu-Plg) (24), named for its N-terminal glutamate, maintains a compact, closed conformation. Upon limited feed-back loop proteolytic processing by plasmin on cellular or ECM surfaces, the PAN domain is removed, and Lys78 gets exposed at the Nterminus, giving rise to the form termed Lys-plasminogen (Lys-Plg), which adopts the more relaxed, activation-prone state (25) (**Figure 2A**). This arrangement typically occurs in an environment that is disposed to activation. The binding of Plg to the cell surface or to ECM via the lysine binding site of kringle domains induces a conformational change – a conversion from the closed form to the open form, which is accessible for activation of zymogen to active plasmin (26).

Closed quaternary structure of Glu-Plg is maintained by several intramolecular surface contacts connecting non-adjacent domains: PAN is associated with the K4 and K5, SP with the K2 and partially with K4, and the K3-K4 linker interconnects K4 with SP and the activation linker. During domain association, the lysine-binding sites (LBSs) in K2, K4, and K5 are occupied by the side chains of interacting domains (**Figure 2B**). Interestingly, chloride ions bind to the anion-binding sites of K2 and K4 LBSs, forming interdomain contacts that are important for the closed Plg conformation. Only LBS in K1 is solvent-accessible and can interact directly with the cell surface or the ECM (23, 27). Virtually all scissile bonds in Glu-Plg are inaccessible in Plg closed form and require a degree of opening of the molecule to be cleaved.

After activation, the newly created amino terminus at Val562 rotates and forms a tight salt bridge with the side chain of Asp740, thereby creating a functional enzyme active site. Structurally, proteolytic activation of Plg to Pm requires at least two steps: 1. Opening the Plg structure to expose the activation loop and 2. Reconstruction of the active cleavage site by cleaving the activation loop.

PAN and SP domains are standard globular structures, whereas kringles contain only a short antiparallel beta sheet at the bottom of the LBS pockets (**Figure 2B**); their shape is held together mainly by three pairs of disulfide bonds. It results in them being structurally malleable and flexible, as reflected by the relatively large B-factor values of K3 and K5 in available structures of intact Plg.

## Mechanism of Plg activation

**Figure 3:**
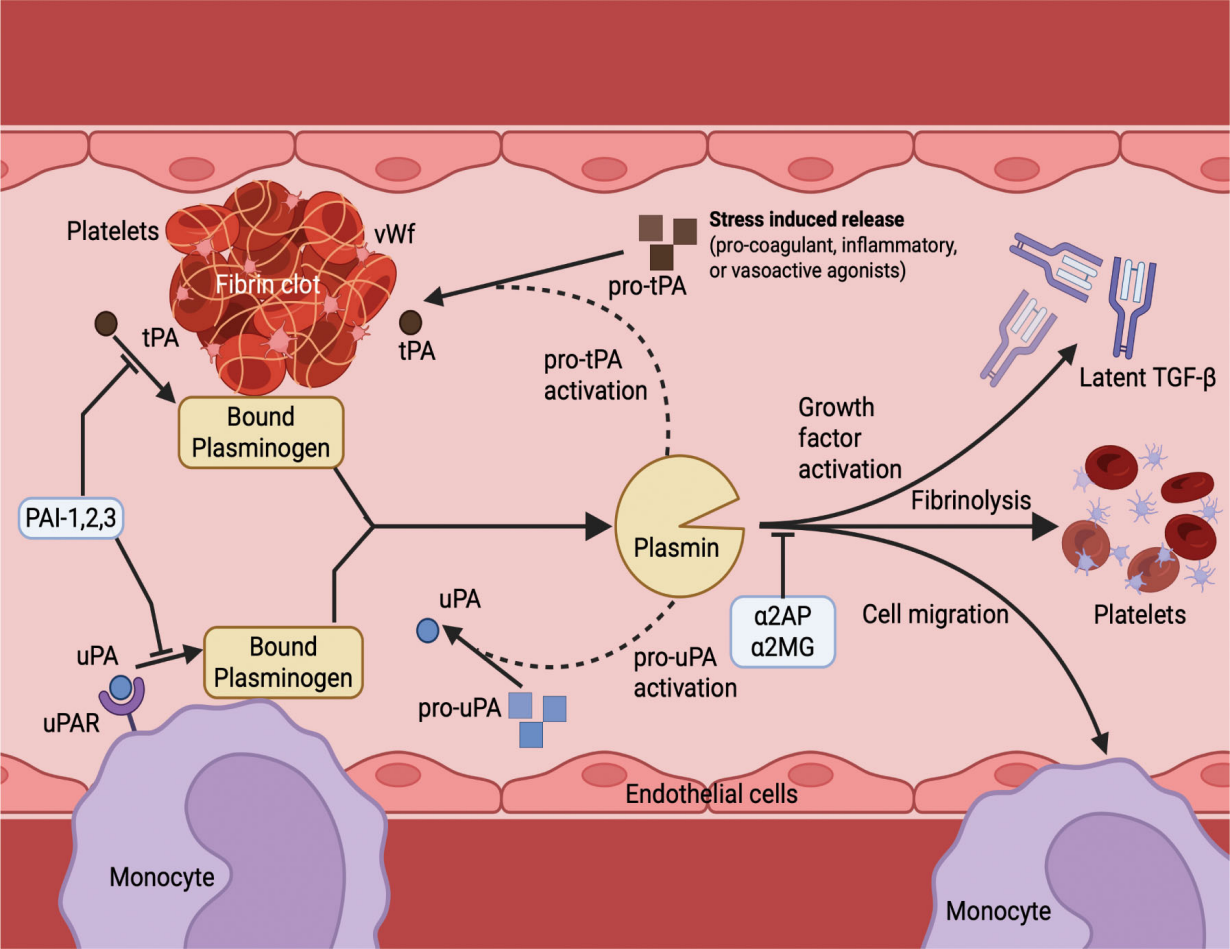
Plg activation pathways. The graphic illustrates the dual pathway of Plg activation. Plg activators are released in pro-active forms and processed to the active Plg activators by plasmin. On fibrin clots, tissue-type plasminogen activator (tPA) binds to fibrin, converting Plg to plasmin, which then degrades the clot. On the cell surface, urokinase-type plasminogen activator (uPA) binds its receptor (uPAR) to activate Plg to plasmin for pericellular proteolysis and cell migration. Plasminogen activator inhibitors (PAI-1) inhibit tPA and uPA, whereas alpha-2 antiplasmin rapidly inactivates free plasmin, preventing excessive fibrinolysis. Figure was created with BioRender.com.

**Figure 4:**
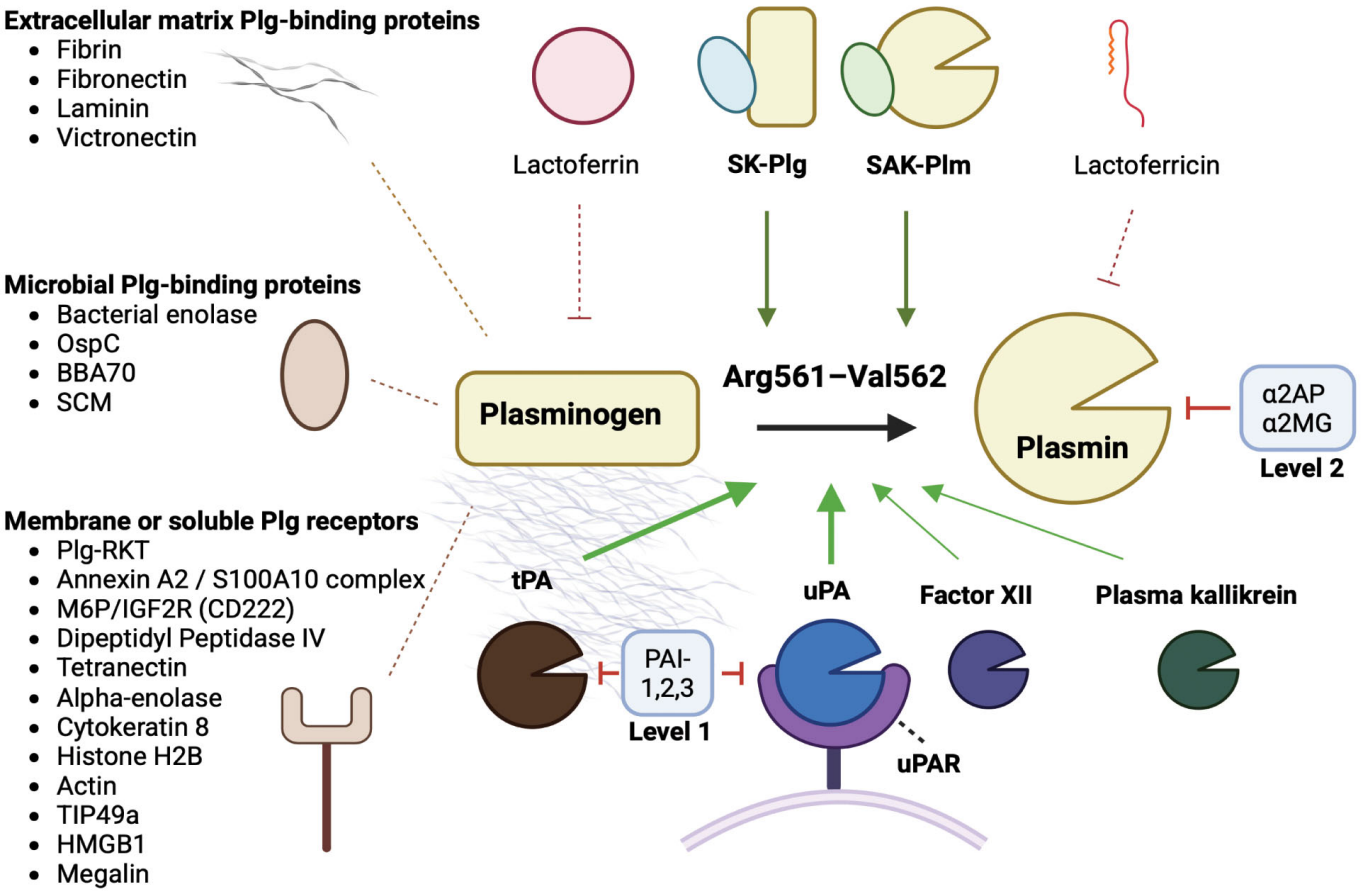
Plg interaction network including regulatory modulators. Schematic overview of proteins interacting with Plg and plasmin (177), including key regulatory components. Plg is converted to Plm by proteolytic cleavage at Arg561–Val562. Direct activators include tPA and uPA, the principal physiological enzymes, with factor XII and plasma kallikrein as additional context-dependent activators. Indirect microbial activators (SK and SAK) promote Plg activation by forming complexes. Activation by tPA and uPA is controlled by plasminogen activator inhibitors (PAI-1, PAI-2, PAI-3), while plasmin activity is primarily inhibited by α2-antiplasmin (α2AP) and α2-macroglobulin (α2MG). Extracellular matrix components, microbial Plg-binding proteins, and cellular receptors facilitate Plg localisation and surface recruitment. Solid arrows indicate activation, inhibitory lines denote suppression of enzymatic activity, dashed lines represent binding interactions, and line thickness reflects the relative contribution to Plg activation/inhibition; Abbreviations: SK, streptokinase; SAK, staphylokinase; OspC, outer surface protein C; BBA70, Borrelia burgdorferi antigen 70, SCM, Streptococcus canis M-like Protein; Plg-Rkt, plasminogen receptor (Rkt); α2MG, alpha-2-macroglobulin; α2ap, alpha-2-antiplasmin; HMGB1; high mobility group box 1, M6P/IGF2R, mannose-6-phosphate/insulin-like growth factor 2 receptor; PAI; plasminogen activator inhibitor-1; tPA; tissue plasminogen activator; uPA, urokinase-type plasminogen activator; TIP49a; TATA-binding protein-interacting protein 49 kDa, isoform A. Figure was created with BioRender.com.

Activation of Plg to plasmin was observed in many places, including urine, blood, and vascular tissue. The first identified Plg activator was purified from urine and was hence named urokinase, also known as the urokinase-type plasminogen activator (uPA). Similarly, the Plg activator isolated from tissues was named the tissue-type plasminogen activator (tPA). There are structural and functional similarities between the two. Both are serine proteases with long N-terminal extensions. Common features of these proteases are repetitive regions and modules. Plg activation is a tightly regulated process that does not occur in solution but on surfaces – either on fibrin clots (fibrinolysis/thrombolysis) or on cell surfaces (cell migration) (28, 29) (**Figure 3**, **4**).

### Plg activation on fibrin clots

When blood vessels are wounded, fibrinogen, a terminal component of the coagulation cascade, is converted to fibrin by thrombin, forming fibrin clots to prevent bleeding. When clots are no longer required, they must be dissolved to prevent thrombosis. Then, the fibrinolysis system takes charge. Activation of Plg on fibrin clots is clinically relevant because it enables localised fibrinolysis, preventing widespread bleeding while targeting clot dissolution (30).

In particular, Plg binds to fibrin via LBSs on its kringle domains. Lysine residues in Plg, carrying positive charges, drive specific binding to negatively charged sites on fibrin, enabling targeted clot resolution. This interaction enhances activation efficiency and restricts proteolysis to thrombi. When the Plg molecule is bound to a fibrin clot, it is specifically cleaved by tPA, a serine protease (**Figure 3**). The tPA polypeptide is secreted as a single-chain polypeptide (pro-tPA or sc-tPA) from a limited number of cell types, primarily endothelial cells, vascular smooth muscle cells, neurons, and neural crest-derived cells. Upon stimulation by pro-coagulant, inflammatory, or vasoactive agonists, tPA and von Willebrand factor (vWf) are released from human umbilical vein endothelial cells (HUVEC). It has been proposed that tPA be released from Weibel–Palade bodies (WPB), which also store von Willebrand factor (31), or from separate storage particles (32). A revised model of t-PA secretion supports the latter model (33).

**Figure 5:**
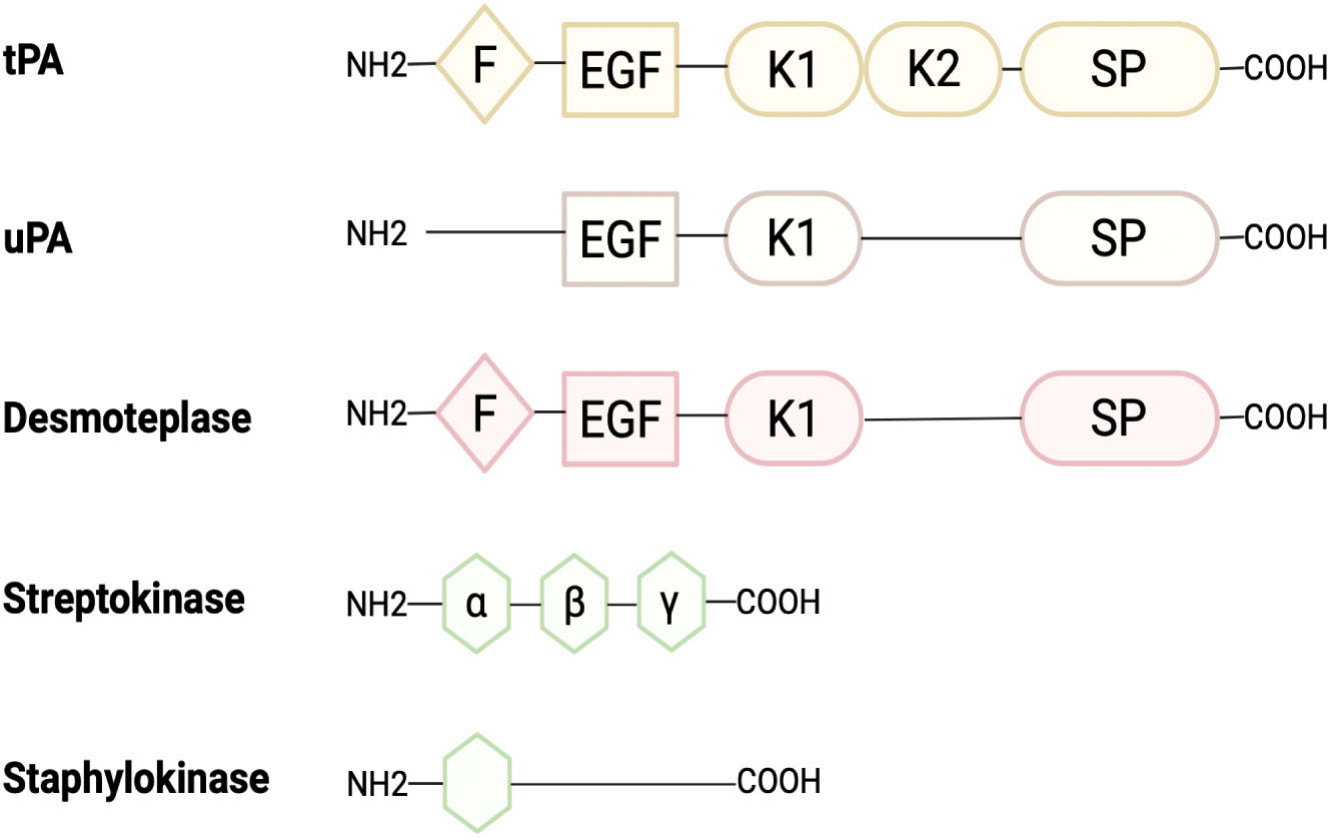
Comparison of domain organisation of eukaryotic and prokaryotic Plg activators. The figure shows the individual domains of the eukaryotic proteins tPA, uPA, and desmoteplase, including the finger domain (F), epidermal growth factor–like domain (EGF), kringle domains (K1, K2), and the serine protease domain (SP). For the prokaryotic proteins, the α, β, and γ domains (streptokinase) and the single-domain organisation (staphylokinase) are shown. Figure was created with BioRender.com. .

In central nervous system (CNS) neurons, tPA is also stored in granules and can be released calcium-dependently by membrane depolarisation. The secretion represents a regulatory step in tPA activity and may contribute to the mechanism by which tPA mediates neuronal plasticity in the brain (34). At the N-terminus, tPA contains the fibronectin type-1 (F1) module. Fibronectin contains 12 repeats of the F1 module. Such a module was also found in blood coagulation factor XII and closely related HGFA (28). Next, tPA contains two repeated kringle modules (26) (**Figure 4**, **5**), of which the second contains lysine-binding sites. This module appears to play an important role in tPA binding to fibrin. In contrast to most zymogens, single-chain pro-tPA exhibits unusually high intrinsic catalytic activity, which is further enhanced upon plasmin-mediated proteolytic cleavage to the two-chain active form. tPA binds to fibrin with high affinity, forming a ternary complex (fibrin–Plg–tPA). This considerably increases the efficiency of Plg activation to plasmin. In the presence of fibrin, tPA-mediated activation increases by three orders of magnitude (29). In particular, tPA cleaves Plg at Arg561-Val562 to generate plasmin. The resulting two chains are connected with two disulfide linkages with a heavier chain moiety at the N-terminus. Plasmin then degrades fibrin, leading to clot dissolution (35).

It has been suggested that fibrinolysis may involve complementary actions of tPA and uPA (36). In this proposed model, tPA initiates fibrin-bound Plg activation, whereas uPA, which does not bind to fibrin, can more readily penetrate deeper layers of the clot and thereby contribute to further Plg activation and plasmin generation. Available clinical data provide support for this coordinated mode of action (37).

### Plg activation on the cell surface

The Plg activation on the cell surface is inevitable for cell migration through tissue barriers. It is clinically relevant because it generates localised pericellular proteolysis, enabling processes such as tissue remodelling, wound healing, inflammation resolution, and angiogenesis, as well as pathological events such as cancer invasion and bacterial dissemination. In addition, plasmin activity on the cell surface conditions the proteolytic activation of growth factors, proteases, or complement components, as well as the proteolytic processing of cell surface receptors, to produce or remove binding sites (28, 38).

Plg activation on cells is mediated primarily by uPA bound on its receptor – the urokinase-type plasminogen activator receptor (uPAR, CD87) (**Figure 3**, **4**). In cell-mediated proteolysis, uPA is the central Plg activator (39). Various migratory cells, such as immune cells, epithelial, endothelial cells, fibroblasts, and many other cell types involved in tissue remodelling or inflammation, produce uPA (40, 41). The expression of uPA is elevated in activated leukocytes and migrating keratinocytes (28). In many biological situations, leukocytes are an important source of uPA. In neutrophils, uPA is stored in specific granules and also in easily mobilisable secretory vesicles. In both compartments, uPAR was also detected, suggesting that it may be occupied on the cell surface prior to exocytosis (28). The uPA polypeptide is secreted as the inactive single-chain 53-kDa glycoprotein, termed pro-urokinase (pro-uPA), which consists of three functionally independent regions: the epidermal growth factor-like domain (EGF-like domain), the kringle domain, and the serine protease region (**Figure 4**, **5**). The N-terminal EGF-like domain of uPA is specifically responsible for its high-affinity interaction with uPAR. Upon binding to uPAR, pro-uPA is cleaved by plasmin, cathepsins, or components of the coagulation system and kallikrein-kinin system (KKS), e.g., factor XIIa, plasma kallikrein, into the enzymatically active two-chain high molecular weight HMW-uPA, interlinking various proteolytic systems (42–45). Further cleavage of HMW-uPA yields the low-molecular-weight LMW-uPA, which is shortened by the amino-terminal fragment (ATF). LMW-uPA then activates Plg into serine protease plasmin (46). The kringle domain of uPA lacks a typical LBS but has a characteristic basic sequence motif with affinity for heparin (47). The C-terminal catalytic domain shares high sequence similarity with other members of the Plg activation system and also with archetypal family members trypsin and chymotrypsin (28). Upon binding to uPAR, the active uPA cleaves the cell-bound Plg with high specificity at Arg_561_-Val_562_. The action of uPA is a typical example of reciprocal zymogen activation: uPA catalyses the activation of Plg, and the generated plasmin, in a positive feedback loop, activates pro-uPA. In contrast, uPAR *per se* might serve as a plasmin substrate, leading to the removal of the uPA-binding site. This creates a negative feedback loop in Plg activation at the cell surface (48).

The human uPAR (49) is a 55–60 kDa, heavily glycosylated cell-surface glycoprotein anchored in the plasma membrane via a glycosylphosphatidylinositol (GPI) moiety. uPAR is expressed by T-cells, NK cells, monocytes, and neutrophils, as well as by non-hematopoietic cells, including vascular endothelial cells, fibroblasts, smooth muscle cells, keratinocytes, placental trophoblasts, hepatocytes, and other migratory cells. Nevertheless, it was shown that the chimeric pro-uPA, when anchored to the cell surface via a GPI anchor, generates plasmin with the same characteristics as uPAR-bound uPA (50). In addition, the lysine-dependent interaction between uPA and Plg is necessary for the assembly of cell-surface Plg activation complexes (51). Therefore, it seems that uPAR is not directly involved in the complex formation leading to Plg activation, but rather, via interactions with other partner molecules, uPAR is crucial for localising Plg activation for cell migration, growth factor activation, or internalisation (6, 52–54). Notably, in addition to uPA and tPA, plasma kallikrein has been proposed to contribute to Plg activation, particularly during skin wound healing (55).

## Knock-out studies of the Plg activation system

Plg deficiency in mice results in high mortality, wasting, and further severe disorders due to severe thrombosis, fibrin deposition in the liver, lungs, and other organs (56), and impaired cardiac wound healing (57). While most of these effects are rescued by concomitant fibrinogen deficiency (58), certain phenotypes — particularly in the central nervous system — persist, indicating that Plg has additional fibrin-independent roles beyond fibrinolysis (**Table 1**).

**Table 1:** Mice phenotypes in the knock-out of the Plg activation system’s components.

Knockout model	Viability	Key phenotype	Original references
Plasminogen (Plg−/−)	Postnatal lethal	Severe thrombosis, fibrin deposition in liver, lungs, and other organs; retarded growth; impaired wound healing.	(56, 57)
Fibrinogen (Fib−/−)	Viable	Severe bleeding disorders, impaired platelet aggregation, prolonged bleeding after injury; not embryonically lethal in most genetic backgrounds.	(213, 214)
Double Fibrinogen + Plasminogen (Fib−/−; Plg−/−)	Viable	No spontaneous thrombosis, decreased viability, wasting disease, extravascular fibrin deposits.	(58)
tPA (tPA−/−)	Viable	Impaired thrombolysis, fibrin accumulation in organs (e.g., lungs, liver), reduced synaptic plasticity.	(39)
uPA (uPA−/−)	Viable	Impaired tissue remodeling, wound healing, and cell migration; reduced tumor metastasis and invasion.	(39)
uPAR (uPAR−/−)	Viable	Defects in neutrophil recruitment, tissue reorganization, reduced tumor metastasis and invasion.	(61)
Combined tPA−/−; uPA−/−	Perinatal lethal	Widespread fibrin deposition, severe organ failure due to uncontrolled fibrin accumulation.	(39)

Plg deficiency in a genetic mouse model of experimental nephrotic syndrome using the conditional podocin knockout model does not prevent sodium retention, indicating that other urinary serine proteases—not plasmin alone—are critical for this process (59).

The uPAR-/- mice were reported to be viable but to exhibit delayed and weaker leukocyte recruitment in response to inflammatory stimuli (60, 61). Similarly, genetic knock-outs of uPA and tPA, although they led to partially impaired pathways in thrombolysis, cell migration and tissue remodelling, were not lethal. The uPA-/- mice have impaired lymphocyte-mediated host defence against defined pathogens (62) and exhibit extensive fibrin deposition, with associated effects on growth, fertility, and survival (39). On the other hand, overexpression of uPA leads to impaired clotting functions with the risk of severe bleeding (63). tPA knockout mice show reduced synaptic plasticity in hippocampal neurons (64), whereas transgenic mice overexpressing tPA show an increased and prolonged hippocampal long-term potentiation (65).

The non-lethal phenotype of mice with knockouts of individual serine proteases in the fibrinolytic system can largely be explained by functional redundancy among these proteases and other compensatory mechanisms. Indeed, the combined KO of both tPA and uPA led to perinatal death due to widespread fibrin deposition, thrombosis, and organ failure (39).

## Regulation of Plg activation

In contrast to very specific Plg activators, plasmin *per se* has a broad substrate spectrum. To avoid rampant proteolysis, both Plg activation and intrinsic plasmin must be controlled (**Figure 3** and **4**). The first level of control is guaranteed by the structural flexibility of the Plg molecule. Plg exists in two major conformations. In a soluble state, i.e. in the circulation or in the extracellular milieu, Plg shapes in a closed form: The N-terminal PAp domain and kringle domains are folded in a way that masks the activation site (Arg561-Val562), making it less accessible to activators like tPA and uPA, and more vulnerable to its inhibitors (**Figure 2B**). When bound to the surface, Plg figures in an open form: The PAp and kringle domains undergo a conformational change, exposing the activation site and enhancing binding to fibrin or cell surface receptors. This form is more readily activated to plasmin (23, 26). Thus, free Plg cannot be activated *in vivo*.

### Regulation by inhibitors

The process of Plg activation is physiologically primarily regulated by Plg activator inhibitors (**Figure 4**), which belong to the serpin (serine protease inhibitor) superfamily. This group of inhibitors exhibits a conserved structure and adopts a conformation that is required for inhibition activity. Serpins inhibit target proteases by an irreversible mechanism, forming a covalent ester linkage between the protease and the reactive central loop of the inhibitor (66). Plasminogen activator inhibitor-1 (PAI-1) inhibits both tPA and uPA, preventing the conversion of Plg to plasmin. PAI-1 forms a 1:1 complex with tPA/uPA, blocking their enzymatic activity (67). PAI-1 is expressed in many cell types in a highly regulated manner, and low concentrations can be found in plasma, too. PAI-1-mediated inhibition is particularly effective at the cell surface. Once formed, the uPAR:uPA: PAI-1 complex is immediately internalised by the low-density lipoprotein receptor-related protein/a2-macroglobulin receptor (LRP/a2MR, CD91) and directed to lysosomes for degradation; unengaged uPAR recycles back to the cell surface (68, 69). However, there is also an additional mechanism that can internalise uPAR independently of the CD91 pathway (70). Plasminogen activator inhibitor-2 (PAI-2) belongs to a different subclass of serpins, has a much more restricted expression pattern than PAI-1, and is found primarily in monocytes and the placenta. It lacks a signal sequence and is poorly secreted, suggesting an intracellular role rather than an inhibitor of Plg activators. It is a very poor inhibitor of tPA (more than 1000-fold lower than PAI-1) and only a moderate inhibitor of uPA (67, 71). Plasminogen activator inhibitor-3 (PAI-3, Protein C Inhibitor) inhibits both Plg activators and activated protein C, but its role in plasmin inhibition is less direct (72).

The next level is provided by direct plasmin inhibitors (**Figure 4**). Alpha2-Antiplasmin (α2AP) is the fastest and highly specific inhibitor of plasmin in circulation. It forms a 1:1 stoichiometric, irreversible complex with plasmin, which is inactivated within the complex (73). Free plasmin in circulation is quickly neutralised by α2AP, preventing unwanted systemic fibrinolysis. Any free plasmin that remained uninhibited by α2AP would be neutralised by α2-macroglobulin (α2M). This non-serpin, broad-spectrum protease inhibitor traps plasmin (and other serine proteases, such as tPA, uPA, and both plasma and tissue kallikreins) within its cage-like structure. Unlike α2AP, α2M does not form a covalent bond; instead, it physically entraps the protease, preventing it from accessing substrates. It is less specific and acts as a “backup” inhibitor when α2AP is overwhelmed or saturated (53, 74). In plasma, Plg circulates in slightly lower concentrations (~0.2 g/L; 2 µM) than α2M (~1.5–4 g/L; 2–5 µM) and fibrinogen (2–4 g/L; 5–12 µM). This balance ensures that any plasmin generated is quickly inhibited, preventing systemic proteolysis (75, 76). For comparison, plasma concentration of albumin is much higher (~35–50 g/L; 500–800 µM) (77).

Breast milk glycoprotein lactoferrin was shown to inhibit Plg activation via blocking Plg binding to uPA (78). In addition, lactoferricin, the N-terminal bioactive peptide generated from lactoferrin upon digestion, was shown to directly inhibit plasmin and other homologous serine proteases, such as elastase or TMPRSS2, the latter of which is critically involved in the proteolytic priming of SARS-CoV-2 (79).

In addition to the endogenous inhibitors of Plg activation and plasmin activity, the pharmacological modulation of fibrinolysis can be provided with lysine analogues such as tranexamic acid (TXA) and ϵ-aminocaproic acid (EACA), which reversibly bind to LBSs within the kringle domains of Plg, thereby preventing its recruitment to fibrin surfaces and subsequent activation to plasmin, which suppresses fibrinolysis. They are used clinically as antifibrinolytic agents in conditions such as trauma-induced blood loss, postpartum haemorrhage, or surgical blood loss (80–83).

Also, synthetic inhibitors are used in medicine and research. The diuretic drug amiloride selectively inhibits uPA activity but not tPA (84). Relatively efficient tPA inhibitors include some plant–derived Kunitz inhibitors (85, 86). Aprotinin is a well-known direct inhibitor of plasmin that forms a very stable noncovalent complex with the protease (87).

### Regulation by receptors

Surfaces, either the cell or ECM, play a central role in controlling Plg activation and plasmin activity. The high affinity of tPA for fibrin ensures that plasmin is generated only where clots form, minimising the risk of bleeding. Furthermore, tPA is most active when bound to fibrin, ensuring localised clot lysis and minimising systemic fibrinolysis. Next, plasmin, through its activity, exposes additional lysine residues on fibrin, thereby enhancing further Plg binding and activation (48).

Similarly, Plg activation and plasmin activity are confined to the cell surface, preventing systemic proteolysis. Cellular Plg receptors are heterogeneous in nature, and their number on the cell might be rapidly modulated (88). Their common features are: 1) low affinity; 2) high density; and 3) ubiquitous distribution. The low affinity may be essential to limit excessive proteolysis. As plasmin dissociates from the surface, its activity is rapidly neutralised, since only the surface-bound plasmin is protected from the inhibitory plasma environment (89).

## Plg-derived active fragments

Through limited proteolysis of Plg, naturally active molecules may be yielded (**Figure 2A**), namely angiostatin, mini-plasminogen (miniPlg), and microplasminogen (microPlg). Angiostatins are generated through the proteolytic cleavage of Plg by enzymes such as matrix metalloproteinases (MMPs), elastase, and cathepsins, which release the kringle domains 1–4 or 1–3 of Plg (90). Since the lack of the proteolytic domain, these fragments do not convey any proteolytic activity. On the other hand, by binding to endothelial cell surface receptors (e.g., ATP synthase, integrins) and disrupting signalling pathways critical for endothelial cell proliferation, migration, and tube formation, angiostatin fragments act as potent inhibitors of angiogenesis (91, 92). Angiostatin’s anti-angiogenic activity is particularly relevant in tumour suppression, where it inhibits the growth of new blood vessels, thereby limiting tumour progression and metastasis (90, 93).

In contrast to angiostatin, miniPlg is a truncated form of Plg that retains the protease domain together with kringle 5 but lacks the N-terminal pre-activation peptide (94) and other kringle domains. Similarly to angiostatin, it may be generated by plasmin, MMPs, elastase, or thrombin in fibrin-rich environments, such as blood clots, tumours, or inflammatory sites. Once activated to miniplasmin, it exhibits localised fibrinolytic activity (94, 95). This facilitates cell migration, tissue remodelling, and wound repair. Additionally, miniPlg contributes to the regulation of angiogenesis, for example, by inducing endothelial cell apoptosis (6). Together, these Plg-derived fragments highlight the multifaceted role of the Plg/plasmin system in regulating not only fibrinolysis but also angiogenesis.

## Emerging physiological functions of Plg

Plg is constitutively expressed in the liver by hepatocytes (96); notably, Plg expression in hippocampal neurons was detected after excitotoxic injury (97), suggesting that hepatocytes are not the only cells that may produce and secrete Plg. It circulates in plasma at a concentration of 2 µM and is also present in the extracellular space of most tissues (215).

### Matrix-associated Plg system

On fibrin clots, Plg is converted by tPA to plasmin that, in turn, digests fibrin fibres, resulting in clot removal, which prevents thrombosis ultimately (**Figure 3**). Over the past two decades, the field of fibrinolysis has witnessed significant advances in understanding the molecular mechanisms that govern fibrin clot degradation. By means of intravital confocal fluorescence microscopy, Plg appeared to accumulate in the centre of microthrombi during the early phase of microthrombus formation (98). High-resolution imaging has revealed that the regulation of fibrinolysis depends on the nature of fibrin fibres, with tPA-mediated Plg activation being strongly associated with agglomerates in coarse but not fine fibrin (99). Surprisingly, it has been shown that the activated Hageman factor, Factor XII of the coagulation cascade (FXII), not only binds to fibrin, increasing the density and stiffness of the fibrin clot, but also directly converts Plg into plasmin, thereby reducing clot lysis time (100). This clearly indicates that fibrinolytic and coagulation systems do not simply counteract each other but rather that the individual components of the two proteolytic cascades act together to maintain hemostasis.

Recently, the identification of novel biomarkers, therapeutic agents, and fibrinolytic modulators in microRNAs (miRNAs) has further expanded the regulatory network controlling clot dissolution (101). Although miRNAs are packaged into exosomes at very low levels (102), they can exert specific functions in regulating fibrinolysis. For example, the miR-143 and miR-145 were shown to reduce PAI-1 expression in bladder cancer (103). Another study indicates a regulatory role for miR-30b in targeting PAI-1 during inflammatory states and thrombotic conditions (104). In accordance with these notions, in COVID-19 patients, who frequently display rampant coagulation, miRNAs were detected targeting genes involved in the fibrinolytic system (105). Moreover, circulating microRNAs have emerged as promising biomarkers for predicting stroke risk, stroke severity, and clinical outcomes (106). In addition to microRNAs, exosomes carrying native fibrinolytic enzymes, such as tPA or PAI-1, have been suggested as a promising diagnostic and therapeutic tool (107–109). Moreover, exosomes loaded with Plg-binding molecules can directly modulate fibrinolysis and cell migration. For example, S100A10-positive exosomes derived from cancer cells promote their dissemination (110). S100A10 is a Plg receptor that activates Plg in a complex with annexin 2 (111).

Interestingly, the fibrin scaffold loaded with Plg may serve as a drug delivery system for the treatment of chronic diabetic ulcers (112). In addition to fibrin, Plg also binds to other ECM proteins, such as vitronectin, laminin or fibronectin, which may provide a further level of regulation of fibrinolysis (95, 113). In this context, it is notable that a cross-talk mechanism, in which matrix-bound Plg was activated by uPA secreted by cells, was detected (114).

Notably, the above-described evolutionary kinship between Lp(a) and Plg Lp(a) may underlie the atherogenic potential of oxidised Lp(a) and its contribution to thrombotic cardiovascular risk since Lp(a) was shown to compete with Plg for binding sites on fibrin clots, where it may reduce Plg recruitment and impair fibrinolysis (115). On the other hand, exosomes produced by keratinocytes were shown to promote wound healing via pro-fibrinolytic effects of mesoglycan (116).

### Cell-associated Plg system

The structural heterogeneity of Plg receptors, combined with their high density and broad distribution, is consistent with a diversified role of Plg in various biological processes. Plg receptors may bind Plg either via their carboxy-terminal lysine residues recognising lysine-binding kringle domains, or independently of lysines (**Figure 4**, **Table 2**).

**Table 2:** Plg receptors.

Protein	Function	Cell Origin	Lysine Dependency	Reference
Alpha-enolase	Glycolytic enzyme; cell-surface plasminogen receptor; enhances plasminogen activation	Monocytes, macrophages, cancer cells, bacteria	C-terminal lysine	(124, 125)
Cytokeratin 8	Intermediate filament protein; major plasminogen receptor on breast cancer cells	Epithelial cells, hepatocytes, breast carcinoma cells	C-terminal lysine	(126)
TIP49a	Nuclear protein; profibrinolytic plasminogen-binding protein	Monocytoid cells, macrophages	C-terminal lysine	(127)
Plg-RKT	Transmembrane plasminogen receptor; promotes plasminogen activation and macropinocytosis	Macrophages, monocytes, liver cells	C-terminal lysine	(129)
Histone H2B	Nuclear protein; plasminogen receptor on apoptotic cells	Nucleated cells, apoptotic cells	C-terminal lysine	(135)
S100A10	Calcium-binding protein; forms heterotetramer with annexin A2; enhances plasminogen activation	Endothelial cells, monocytes, macrophages	C-terminal lysine (via annexin A2)	(118)
Actin	Cytoskeletal protein; cell-surface plasminogen receptor; promotes plasminogen activation	Adrenal chromaffin cells, astrocytes, platelets	Requires proteolytic exposure of lysine	(138)
Amphoterin (HMGB1)	Heparin-binding protein; enhances plasminogen activation and cell migration	Neurons, macrophages, transformed cells	Lysine-rich N-terminal region	(137)
Annexin II	Calcium-binding protein; coreceptor for tPA and plasminogen; enhances fibrinolysis	Endothelial cells, monocytes, macrophages, cancer cells	C-terminal lysine	(120)
Glycoprotein IIb/IIIa	Integrin; platelet aggregation; fibrinogen binding; potential plasminogen interaction	Platelets	Not directly lysine-dependent	(139)
GP330 (Megalin)	Endocytic receptor; binds plasminogen and plasminogen activator complexes	Renal proximal tubules, type II pneumocytes	Not directly lysine-dependent	(143)
Tetranectin	Plasminogen kringle 4-binding protein; enhances plasminogen activation	Plasma, extracellular matrix, cancer stroma	Lysine-binding site in kringle 4	(144)
Tissue Factor	Initiates coagulation; binds plasminogen; regulates plasminogen activation	Endothelial cells, monocytes, cancer cells	Kringle 1–3 interaction	(140, 141)
Complement C7 Receptor	Plasminogen binding; role in complement activation	Complement-activated cells	Lysine-independent	(142)
Dipeptidyl Peptidase IV	Plasminogen receptor; role in inflammation and cancer	T-cells, epithelial cells, fibroblasts	Lysine-independent	(145)
M6P/IGF2R (CD222)	Mannose 6-phosphate/insulin-like growth factor 2 receptor; plasminogen binding	Fibroblasts, liver cells	Lysine-independent	(148)
Disease-associated Prion	Plasminogen binding; potential role in prion disease pathogenesis	Neurons, prion-infected cells	Unknown	(146)

First, Plg binding to the cellular receptors might potentiate and restrict Plg activation and plasmin activity to a specific spatial location (117). Then, plasmin may facilitate ECM cleavage, activate precursors of growth factors and proteases, or process cell-surface receptors (**Figure 3**). Hence, the particular substrate of the cell surface-associated plasmin depends on the receptor on which Plg was bound. Such a cell-surface milieu then marks the key signature of Plg function.

For example, S100A10, a member of the S100 protein family, forms a complex with annexin A2, creating a highly efficient Plg activation complex on endothelial and cancer cells, thereby driving plasmin-dependent stimulation of other proteases, such as matrix metalloproteinases (MMPs) -2 and -9 (118, 119). The annexin A2–S100A10 heterotetramer is assembled in the cytoplasm upon binding of S100A10 to annexin A2, followed by Src-dependent phosphorylation of annexin A2 at Tyr23, which promotes membrane translocation of the complex. At the cell surface, S100A10 provides a C-terminal lysine for Plg binding, while annexin A2 anchors the complex to phospholipid membranes. In addition, annexin A2-mediated assembly of Plg and t-PA promotes and localises constitutive plasmin generation on the blood vessel wall (120). The assembly thus results in a marked amplification of Plg activation and local protection of plasmin from α2-antiplasmin (121, 122). Upon epithelial-mesenchymal transition (EMT), S100A10 and PAI-1 are regulated reciprocally by canonical Smad-dependent TGFβ1 and FOXC2-mediated PI3K signalling, and modulate Plg activation to promote plasmin-dependent matrix remodelling or cancer cell invasion (123).

Alpha-enolase is a glycolytic enzyme on the cell surface of various cell types that binds Plg via the C-terminal lysine residue, facilitating localised plasmin generation and promoting cell invasion and ECM remodelling (124, 125). Similarly, cytokeratin 8 on epithelial and cancer cells binds Plg, enhancing pericellular proteolysis (126). TIP49a, a nuclear protein, also binds to Plg, linking intracellular signalling to the extracellular microenvironment (127). Additionally, Plg interacts with the metalloprotease ADAMTS13 (128) and degrades it, thereby regulating von Willebrand factor (VWF) activity.

The Plg receptor, Plg-RKT, is a transmembrane protein that binds both Plg and uPAR to generate active plasmin during inflammation, wound healing, or lactation. It colocalises with uPAR, enhances uPA-dependent Plg activation, and can also bind tPA to promote tPA-mediated activation. Structurally, Plg-RKT is unique in that both its N- and C-terminal domains are exposed on the extracellular surface, with the C-terminal lysine residue serving as a key anchoring site for Plg (129–134).

Histone H2B, exposed on the surface of stimulated neutrophils, binds Plg and contributes significantly to the proteolytic capacity of the cells (135). Surface exposure of H2B occurs under specific cellular conditions, most prominently during immune cell activation, monocyte-to-macrophage differentiation, and apoptosis, and is mediated by calcium-dependent redistribution of intracellular H2B to the plasma membrane rather than *de novo* synthesis. In addition, H2B surface expression has been observed in tumour cells (133, 136). Similarly, amphoterin is another nuclear protein that may be exposed on the cell surface; via binding Plg at the leading membrane, it facilitates cell migration (137). The cell-surface-expressed forms of actin were also shown to bind and activate Plg, thereby facilitating prohormone processing and inhibiting neurotransmitter release (138).

On platelets, glycoprotein GPIIb/IIIa binds Plg upon thrombin stimulation, linking fibrinolysis with coagulation (139); similarly, at extravascular sites, Plg localisation and activation may be modulated through a high-affinity interaction between kringles 1 through 3 of Plg and the extracellular domain of the tissue factor (140, 141). On the other hand, the binding of Plg to the complement C7 receptor (142) links fibrinolysis to the complement system, potentially focusing plasmin activity on structures tagged by Ab and complement deposition. GP330 (143) and tetranectin (144) have been suggested to modulate Plg activation in kidney diseases and tissue repair. Dipeptidyl peptidase IV (DPP IV, CD26) was also identified as a Plg receptor (145), suggesting a possible physiological role of the Plg system in regulating metabolic pathways. Notably, Plg has also been identified as a binding partner of the disease-associated prion protein (146), suggesting a potential role in neurodegenerative diseases. In this respect, it has been implied that plasmin paucity in the brain might be a primary defect in certain cases of sporadic Alzheimer’s Disease (216).

Again, as on clots, the above-described structural homology between Lp(a) and Plg allows the interaction of Lp(a) with Plg binding sites on the cell surface. In particular, apolipoprotein(a) (apo(a)) component of Lp(a), which contains kringle domains with preserved lysine-binding properties, has been shown to interfere with Plg binding mediated by the PlgRKT, annexin A2, and S100A10 complex, thereby modulating local Plg activation. Subsequently, the Lp(a) binding stimulates its macropinocytosis, which may have clinical relevance since several drugs that inhibit macropinocytosis are in clinical use (147).

On the other hand, Plg binding to the cell surface might also lead to down-regulation of Plg or plasmin, e.g., via internalisation. This functional duality of Plg receptors is represented by the mannose 6-phosphate/insulin-like growth factor 2 receptor (M6P/IGF2R, CD222) (148, 149), which, as mentioned above, is encoded by the gene encompassed in the same locus as the gene for Plg, both in human and mouse. First, M6P/IGF2R mediates latent transforming growth factor beta (LTGF-β) activation by binding miniPlg, leading to apoptosis of HUVECs (6); second, M6P/IGF2R targets bound Plg into lysosomes for degradation (150).

Recently, a series of studies revealed the role of the Plg system in efferocytosis, i.e., the clearance of apoptotic cells by phagocytic macrophages. Namely, it has been shown that Plg, via specific binding to apoptotic cells, yields an “eat-me” signal recognised by IGF2R, resulting in the uptake of dead cells by macrophages (4, 151, 152). On apoptotic cells, exposed annexin A2 might serve as a Plg receptor (120, 153).

As mentioned above, in addition to fibrinolysis, the Plg system also participates in other major proteolytic systems, namely, the contact system of the coagulation cascade, the complement pathways, and KKS. In particular, plasmin directly cleaves the complement components C3 and C5, generating C3a and C5a (anaphylatoxins), which promotes chemotaxis and the formation of the membrane attack complex (MAC) (38). Plasmin-mediated degradation of coagulation factors FV, FVIII, FIX, and FX was observed, potentially leading to downregulation of the coagulation cascade (154). On the other hand, plasmin can initiate the contact system of coagulation by directly activating FXII, also known as Hageman factor (155). Interestingly, FXII may reciprocally mediate the Plg activation (156). The activation of the contact system results in the subsequent activation of KKS and production of bradykinin (BK), a potent vasodilator and permeability agent. In hereditary angioedema (HAE), particularly in C1-INH-deficient forms, this cross-talk becomes pathologically evident as “plasminflammation” — where Plg activation significantly amplifies BK generation and drives angioedema attacks (9, 157). Moreover, a missense mutation in the *PLG* gene (p.Lys330Glu) enables “gain-of-function” Plg activation leading to HAE-like symptoms due to uncontrolled BK production and recurrent angioedema despite normal C1-INH levels (158). This extensive molecular crosstalk allows Plg/plasmin to regulate inflammation and vascular permeability (44, 45, 159, 160).

All these interactions expand the regulatory scope of Plg beyond fibrinolysis, influencing immune responses, cell signalling, apoptosis, efferocytosis, homeostasis and metabolic pathways, with implications for cancer, thrombosis, vascular and neurodegenerative diseases.

## Plg system implicated in pathologies

### Bacterial misuse of Plg activation

Plasmin might be hijacked by several pathogenic bacterial strains to increase their invasiveness and virulence. As a rapidly assembled provisional matrix protein, fibrin constitutes an early line of host defence by restricting bacterial proliferation and thereby limiting microbial dissemination to distant sites. Nevertheless, pathogenic bacterial strains have evolved sophisticated mechanisms to circumvent and degrade this fibrin network, thereby promoting their survival and spread (161). They do so via two mechanisms: either directly utilise the host Plg activation system (e.g., uPA) or employ bacterial devices to activate host Plg.

The former mechanism, i.e., the misuse of the host’s Plg activation system, is applied, for example, by Bacillus or Borrelia species. *Bacillus anthracis* binds both Plg and Plg activators (uPA, tPA) to its surface via bacterial proteins (162). Similarly, *Borrelia burgdorferi* binds host Plg to its surface via its multiple outer surface proteins (e.g., OspC, enolase, BBA70) and recruits host-derived Plg activators to its surface, which then converts surface-bound Plg to active plasmin (163–165).

The latter mechanism is utilised by the bacterial proteins, streptokinase and staphylokinase, which, unlike eukaryotic PAs, lack proteolytic activity and instead function as cofactors that convert host Plg into an active enzyme. Although both eukaryotic and prokaryotic PAs ultimately promote fibrinolysis, their structural organisation, evolutionary origin, and regulatory properties differ profoundly (**Figure 5**). Streptokinase (SK), a ~47 kDa protein secreted by group A, C, and G streptococci (e.g. *Streptococcus pyogenes*), is composed of three structural domains (α, β, γ) and lacks both disulfide bonds and glycosylation (**Figure 5**). It forms a tight stoichiometric complex with Plg or plasmin; specifically, it binds to Plg’s kringle domains, inducing a conformational change that exposes the active site. The SK-Plg complex acts as an active protease, cleaving other Plg molecules at Arg561-Val562 to generate plasmin (166), initiating an autocatalytic amplification loop of plasmin generation (167, 168). Because this mechanism does not rely on fibrin targeting, SK exhibits poor fibrin specificity and a higher risk of systemic fibrinolysis. Similar to SK, staphylokinase (SAK), secreted by certain strains of *Staphylococcus aureus*, can specifically activate the host’s Plg (169). SAK operates through a closely related but mechanistically more restricted pathway. It also lacks intrinsic enzymatic activity and functions by forming a complex with plasmin. However, unlike SK, productive activation by SAK typically requires preformed plasmin rather than Plg alone. Structural studies have revealed that the SAK–plasmin complex behaves as a proteinase–cofactor assembly that efficiently activates fibrin-bound Plg, thereby conferring a degree of fibrin selectivity absent in streptokinase (170–172).

Moreover, streptolysin O, a key cytolytic toxin produced by Group A Streptococcus, binds directly to Plg and accelerates its conversion to plasmin by both SK and host tPA (14). Additionally, the binding of both human Plg and miniPlg has been reported, specifically to the novel M-like protein designated SCM (S. canis M-like protein), which mediates the virulent fibrinolytic activity of *Streptococcus canis* (173).

Plasmin, hijacked by bacteria, not only helps bacteria disseminate by degrading ECM components (e.g., fibrin, fibronectin, laminin) but also enables them to evade the immune system by degrading complement proteins (e.g., C3b, C5) and antibodies (162). Moreover, although plasmin activity is indispensable for immune cells’ migration and thus beneficial in inflammation, by mediating the extensive production of pro-inflammatory cytokines, it can also contribute to tissue destruction in sepsis (174).

On the other hand, owing to their highly specific effects, both SK and SAK have been used as therapeutic agents for thrombolytic therapy in treating acute myocardial infarction, stroke, deep vein thrombosis, and pulmonary embolism (175, 176). Because of their capacity to activate Plg, both SK and SAK have been explored and applied as thrombolytic drugs for the treatment of acute thrombotic events (177). SK has been widely used for decades. The combination of its low production cost and good clinical performance has made it widely available and used globally (167). However, its limited fibrin specificity, leading to systemic Plg activation, undermines this advantage, and SK is being used less frequently in clinical practice. By contrast, SAK demonstrates markedly greater specificity for fibrin-bound Plg, leading to plasmin generation localised at the site of the clot and much lower systemic Plg activation (177). SAK, like SK, can bind Plg; however, the SAK-Plg complex is catalytically inactive (178). Instead, SAK exhibits substantially higher affinity for plasmin, and it is this SAK-plasmin complex that mediates subsequent Plg conversion. This mechanistic distinction underlies its more targeted fibrinolytic profile. Interestingly, after nearly four decades in which alteplase (recombinant form of tPA) remained the only approved treatment for stroke and with tenecteplase (recombinant tPA mutant) introduced only recently (179), SAK is now emerging as a new thrombolytic candidate for stroke therapy (180).

The profound structural differences between the eukaryotic and bacterial PAs reflect their distinct biological roles. Vertebrate PAs evolved within the tightly regulated hemostatic system, where spatial and temporal control of fibrinolysis is essential. Their multidomain architecture enables fine regulation through fibrin binding, receptor interactions, and inhibitor sensitivity. In contrast, bacterial activators evolved as virulence factors that help pathogens breach fibrin barriers and disseminate within host tissues. Their minimalist cofactor strategy provides rapid and potent plasmin generation but at the expense of specificity and immunogenicity. Despite their divergent origins, both groups converge functionally on the same biochemical endpoint-the generation of plasmin and dissolution of fibrin. This convergence highlights the strong evolutionary pressure to exploit the host Plg system. From a therapeutic standpoint, understanding these relationships provides a rational framework for the design of next-generation thrombolytics. Current protein engineering efforts often aim to combine the high fibrin specificity and low immunogenicity of human tPA with the favourable pharmacokinetic or activation properties observed in desmoteplase (181), a recombinant form of the alpha-1 isoform of vampire bat’s salivary Plg activator (**Figure 5**) or bacterial activators. Such hybrid strategies may ultimately yield thrombolytics with improved efficacy, safety, and clot selectivity.

In summary, Plg activators exemplify two contrasting evolutionary solutions to fibrinolysis. Eukaryotic PAs represent homologous multidomain serine proteases whose functional diversity arose through domain remodelling within the vertebrate lineage. SK and SAK, in contrast, are structurally simple bacterial cofactors that hijack host Plg via protein-protein interactions. Appreciating these structural and evolutionary relationships is essential for understanding their biological behaviour and for guiding the rational engineering of improved thrombolytic therapeutics.

### Viral priming by plasmin

Viral priming is, in principle, opening cell gates to viruses by means of the host’s proteolytic system. Namely, plasmin was shown to enhance viral infectivity, dissemination, and pathogenesis by cleaving viral envelope proteins, activating latent viruses, or degrading extracellular barriers. This mechanism is particularly relevant for enveloped viruses, including influenza (182, 183), HIV (184), herpes simplex virus (185), and SARS-CoV-2 (79, 186), in which plasmin-mediated cleavage of viral glycoproteins facilitates viral entry, fusion, and spread.

In the context of SARS-CoV-2, additional evidence for plasmin-mediated viral priming was provided by Hou et al. (187). By analysing single-cell transcriptomes from human respiratory tissues, the authors identified co-expression of *PLAU* (uPA), SCNN1G (γ-subunit of the epithelial sodium channel, γ-ENaC), and ACE2 (angiotensin-converting enzyme 2, the primary cellular receptor for SARS-CoV-2) in several airway and alveolar epithelial cell types, indicating that the components required for local plasmin generation and proteolytic processing are present within the same cellular niche. In bronchoalveolar lavage samples from patients with moderate to severe COVID-19, uPA was markedly upregulated, and SARS-CoV-2 infection similarly induced uPA. Additionally, in epithelial cell lines, SARS-CoV-2 infection also induced Plg expression. Functionally, plasmin cleaved the SARS-CoV-2 spike protein and enhanced viral entry into human ACE2-expressing cells, whereas inhibition with PAI-1 reduced spike cleavage and viral uptake. These observations suggest that during infection, increased uPA activity and plasmin generation may be diverted from physiological substrates such as ENaC towards spike priming, thereby facilitating SARS-CoV-2 entry.

Notably, lactoferrin, which has been shown to be a natural inhibitor of plasmin (78), can block *in vitro* infection with herpes simplex virus (188, 189) and SARS-CoV-2 (79, 190). Interestingly, angiostatin, a Plg-derived active fragment, reduced the cellular entry of multiple SARS-CoV-2 variants by interfering with spike protein proteolysis (191). Notably, in accordance with its antifibrinolytic properties, lactoferrin has recently been identified as a physiological modulator of gestational hypercoagulability, making it a potential therapeutic target for pregnancy-associated venous thromboembolism (192). Whether elevated lactoferrin during pregnancy protects pregnant women against Covid-19 remains to be determined by future studies (193).

### Plg system in cancer

It has been demonstrated by Albert Fischer as early as 100 years ago that cancer cells, unlike healthy cells, produce significantly larger amounts of plasmin (194). Thus, the Plg system has been well recognised as a therapeutic target. Nevertheless, tumour cells for their growing and metastasising apply in principle the same molecular mechanisms as are harnessed in physiological Plg activation (195), which makes Plg-targeting therapy difficult.

Recently, the relevance of the Plg system to humans’ evolutionary vulnerability to cancer has been described. As mentioned above, Wamba et al. identified that, unlike non-human primates, humans possess a Pro153-Ser153 amino acid substitution in Fas Ligand (FasL), a critical immune protein used by T-lymphocytes to kill transformed cells (3). This evolutionary change renders human FasL uniquely susceptible to plasmin-mediated cleavage, particularly in plasmin-rich tumour microenvironments. When plasmin cleaves FasL, it diminishes the cancer-killing function of activated T cells. Thus, therapeutic strategies combining plasmin/PAI-1 inhibitors with immunotherapies hold particular promise for future treatment of aggressive and resistant tumours. The P153S mutation is likely an evolutionary tradeoff that supported larger human brain development by reducing neural progenitor apoptosis, but at the cost of increased cancer vulnerability (3).

Interestingly, as with the plasmin-FasL axis, plasmin also modulates the immune checkpoint TIGIT by proteolytically shedding its ectodomain. In *rhesus macaques*, TIGIT is efficiently shed from T cells by plasmin, potentially enhancing their antitumor immunity. In contrast, human TIGIT is less affected by plasmin. This species difference might also explain the low translational efficiency of anti-TIGIT therapies into human medicine (11).

However, the interconnection of the fibrinolytic system’s components with signalling and other proteolytic cascades makes this therapeutic strategy more complex (**Table 3**). For example, the role of PAI-1 in cancer has been extensively elucidated. While expected to inhibit invasion by blocking plasmin generation, PAI-1 instead promotes tumour progression by inducing programmed death-ligand 1 (PD-L1) expression, thereby creating an immunosuppressive microenvironment. Blockade of PAI-1 significantly reduced immunosuppressive cells, increased cytotoxic T-cell infiltration, and enhanced tumour regression (196–198).

**Table 3:** Therapeutic anti-tumour intervention studies associated with the Plg system.

Target	Therapeutic strategy	Disease	Model system	Reference
PAI-1	The anti-PD-1 antibody nivolumab and the PAI-1 selective inhibitor TM5614	Non–Small Cell Lung Cancer	Mouse	(198)
PAI-1	The selective inhibitor MDI-2268	Glioblastoma	Cell lines + mouse	(199)
PAI-1	TM5614 + nivolumab	Unresectable malignant melanoma, non-small cell lung cancer	Clinical (Phase II trial)	(200, 203)
PAI-1	TM5614 + tyrosine kinase inhibitors	Chronic myeloid leukemia	Clinical (Phase II trial)	(201)
PAI-1	TM5614 + paclitaxel	Taxane-resistant cutaneous angiocarcinoma	Clinical (Phase II trial)	(202)
uPAR	Humanized monoclonal antibodies huATN-658	Breast cancer	Cell lines + mouse	(208)
uPAR	Antibody drug conjugate FL1-PNU	Pancreatic ductal adenocarcinoma	Cell lines	(209)
uPARAP (Endo180)	Antibody drug conjugates	Soft tissue sarcomas	Cell lines + mouse	(206, 207)

Inhibition of PAI-1 with the selective inhibitor MDI-2268 reduced glioblastoma (GMB) cell proliferation. In GBM cells, PAI-1 was also involved in autophagy-related processes, and its secretion was partially dependent on autophagy (199). PAI-1 showed promise as a therapeutic target, particularly in skin malignancies. Two completed clinical trials have investigated the PAI-1 inhibitor TM5614. The first evaluated TM5614 in combination with nivolumab in unresectable malignant melanoma and showed promise in anti-PD-1-refractory patients (200). The second tested TM5614, together with tyrosine kinase inhibitors, in chronic myeloid leukaemia, demonstrated improved responses compared with TKI therapy alone (201). Additionally, two ongoing studies are evaluating TM5614 further. A phase II trial combining TM5614 with paclitaxel in taxane-resistant cutaneous angiosarcoma (202), and another evaluating TM5614 with nivolumab as a third-line therapy for advanced non-small cell lung cancer (203). TM5614 is also being evaluated in a phase II trial investigating its safety and potential efficacy in patients with COVID-19 pneumonia (204).

Humanised monoclonal antibodies against uPAR (huATN-658) significantly reduced breast cancer primary tumour growth and bone metastases in preclinical studies. Or, first-in-class antibody-drug conjugates targeting uPARAP (urokinase plasminogen activator receptor-associated protein, also known as Endo180) have shown excellent efficacy in patient-derived xenografts of soft tissue sarcomas. Clinical trials of uPA inhibitors and uPAR antagonists have entered early-phase testing, though definitive confirmation in humans remains pending (205–208). Additionally, uPAR has been shown as an ADC therapy target for pancreatic ductal adenocarcinoma (PDAC). In aggressive PDAC models, the anti-uPAR ADC FL1-PNU demonstrated potent, targeted antitumor effects (206, 207, 209).

Recently, it has been shown that plasmin is implicated in intracellular signalling pathways driving tumour progression. In hepatocellular carcinoma, plasmin promotes invasion and metastasis by activating the PI3K/AKT/mTOR signalling axis via CXCR4. Importantly, inhibition of Plg activation using the TXA reduced tumour cell invasion and metastasis *in vitro* and limited tumour progression *in vivo*. Notably, chemotherapy increased plasmin expression and metastatic potential, whereas plasmin inhibition attenuated these effects, suggesting that combining chemotherapy with plasmin inhibitors may improve patient outcomes (210).

### Plg system in amyloidosis

Based on the documented neurotoxicity of β-amyloid (Aβ), several therapeutic strategies to reduce Aβ levels in the brain have been developed (217). A number of proteases have been implicated in the proteolytic Aβ clearance, including plasmin (218). It has been suggested that plasmin deficiency in the brain could lead to amyloid aggregation (216). Nevertheless, depletion of Plg in the plasma of an Alzheimer’s disease (AD) mouse model reduced plaque deposition, whereas an increase in plasmin activity through α2AP antisense oligonucleotide treatment exacerbated the brain’s immune response (211). Similarly, plasmin activity has been shown to promote amyloid deposition in a transgenic model of human transthyretin amyloidosis (212). Thus, the role of the Plg system in plaque formation and resolution is complex.

## Conclusion

The Plg system is best known for its role in fibrinolysis, counterbalancing the coagulation system; however, beyond its canonical role in fibrinolysis, the Plg system orchestrates a complex network of processes and engages in extensive crosstalk with cellular receptors and extracellular matrix components. Thus, rather than a sword, though double-edged, Plg should be viewed as a Swiss army knife. It is an evolutionarily and structurally complex molecule, reflected in its integral role in maintaining physiological homeostasis. On the other hand, when dysregulated or misused, the Plg system contributes to pathological processes, such as infections, cancer, or neurodegenerative diseases.

Understanding its molecular intricacies opens avenues for novel diagnostics and therapies, ranging from anti-cancer treatments to fibrinolytic modulators and anti-viral strategies. Future research should prioritise translating these mechanistic insights into clinical practice, particularly in oncological, vascular, and infectious diseases, to fully exploit components of the Plg system as therapeutic targets, biomarkers, or tools.

## Glossary

α2AP: Alpha-2-Antiplasmin
α2M: Alpha-2-Macroglobulin
ACE2: Angiotensin-Converting Enzyme 2
ADAMTS13: A Disintegrin and Metalloproteinase with a Thrombospondin Type 1 Motif, Member 13
ADC: Antibody-Drug Conjugate
ATF: Amino-Terminal Fragment
BBA70: Borrelia burgdorferi Antigen 70
BK: Bradykinin
CD: Cluster of Differentiation
CNS: Central Nervous System
COVID-19: Coronavirus Disease 2019
DPP IV: dipeptidyl peptidase IV
ECM: Extracellular Matrix
EGF: Epidermal Growth Factor
ENaC: Epithelial Sodium Channel
F1: fibronectin type-1
FasL: Fas Ligand
FXII: Factor XII
GMB: Glioblastoma
GPI: Glycosylphosphatidylinositol
HGFA: hepatocyte growth factor activator
HMGB1: High Mobility Group Box 1
HMW-uPA: High Molecular Weight Urokinase-Type Plasminogen Activator
K: Kringle Domain
KKS: Kallikrein-Kinin System
KO: Knockout
LBS: Lysine-Binding Site
LMW-uPA: Low Molecular Weight Urokinase-Type Plasminogen Activator
Lp(a): apolipoprotein(a)
LRP: Low-Density Lipoprotein Receptor-Related Protein/a2-macroglobulin receptor
M6P/IGF2R: Mannose 6-Phosphate/Insulin-Like Growth Factor 2 Receptor
MEROPS: Protease Database
microPLG: micro-plasminogen
MMP: Matrix Metalloproteinases
MR: Mannose Receptor
N: Asparagine
NK: Natural Killer
OspC: Outer Surface Protein C
PAI: Plasminogen Activator Inhibitor
PAN: Plasminogen-Apple-Nematode Domain
Pap: Pan-Apple
PD-1: Programmed Cell Death Protein 1
PDAC: pancreatic adenocarcinoma
PD-L1: Programmed Death-Ligand 1
Plg: Plasminogen
Plg-RKT: Plasminogen Receptor KT
Plm: Plasmin
pro-uPA: Pro-Urokinase-Type Plasminogen Activator
pro-tPA: Pro-Tissue-Type Plasminogen Activator
SAK: Staphylokinase
SARS-CoV-2: Severe Acute Respiratory Syndrome Coronavirus 2
SCM: Streptococcus canis M-like Protein
SK: Streptokinase
SP: Serine Protease Domain
TGF-β: Transforming Growth Factor Beta
tPA: Tissue-Type Plasminogen Activator
uPA: Urokinase-Type Plasminogen Activator
uPAR: Urokinase-Type Plasminogen Activator Receptor
uPARAP: Urokinase Plasminogen Activator Receptor-Associated Protein
V: Valine
VWF: Von Willebrand Factor
γ-ENaC: γ-subunit of the epithelial sodium channel
